# The DNA load of six high-risk human papillomavirus types and its association with cervical lesions

**DOI:** 10.1186/s12885-015-1126-z

**Published:** 2015-03-05

**Authors:** Luisa Del Río-Ospina, Sara Cecilia Soto-De León, Milena Camargo, Darwin Andrés Moreno-Pérez, Ricardo Sánchez, Antonio Pérez-Prados, Manuel Elkin Patarroyo, Manuel Alfonso Patarroyo

**Affiliations:** 1 Molecular Biology and Immunology Department, Fundación Instituto de Inmunología de Colombia (FIDIC), Carrera 50#26-20, Bogotá, Colombia; 2 School of Medicine and Health Sciences, Universidad del Rosario, Carrera 24#63C-69, Bogotá, Colombia; 3 Faculty of Natural and Mathematical Sciences, Universidad del Rosario, Carrera 24#63C-69, Bogotá, Colombia; 4 School of Medicine, Universidad Nacional de Colombia, Carrera 45#26-85, Bogotá, Colombia; 5 Mathematics Department, Universidad Pública de Navarra, Pamplona, Spain

**Keywords:** Cervical intraepithelial neoplasia, HR-HPV, HPV DNA load, RT-PCR

## Abstract

**Background:**

Analysing human papillomavirus (HPV) viral load is important in determining the risk of developing cervical cancer (CC); most knowledge to date regarding HPV viral load and cervical lesions has been related to HPV-16. This study evaluated the association between the viral load of the six most prevalent high-risk viral types in Colombia and cervical intraepithelial neoplasia (CIN) frequency.

**Methods:**

114 women without CIN and 59 women having CIN confirmed by colposcopy, all of them positive by conventional PCR for HPV infection in the initial screening, were included in the study. Samples were tested for six high-risk HPV types to determine viral copy number by real-time PCR. Crude and adjusted odds ratios (OR_a_) were estimated for evaluating the association between each viral type’s DNA load and the risk of cervical lesions occurring.

**Results:**

The highest viral loads were identified for HPV-33 in CIN patients and for HPV-31 in patients without lesions (9.33 HPV copies, 2.95 interquartile range (IQR); 9.41 HPV copies, 2.58 IQR). Lesions were more frequent in HPV-16 patients having a low viral load (3.53 OR_a_, 1.16–10.74 95%CI) compared to those having high HPV-16 load (2.62 OR_a_, 1.08–6.35 95%CI). High viral load in HPV-31 patients was associated with lower CIN frequency (0.34 OR_a_, 0.15–0.78 95%CI).

**Conclusions:**

An association between HPV DNA load and CIN frequency was seen to be type-specific and may have depended on the duration of infection. This analysis has provided information for understanding the effect of HPV DNA load on cervical lesion development.

## Background

The main factor for developing cervical cancer (CC) lies in persistent infection by at least one viral type of high-risk human papillomavirus (HR-HPV). Fifteen types of HR-HPV have been described, 99.7% being associated with cases of CC and/or cervical intraepithelial neoplasia (CIN) [1-3]. However, some host and virus related factors modulate such association, i.e. HPV viral load [4,5].

Researchers have thus become interested in HPV viral load. Its association with infection duration has already been described [6,7]. Prior studies have determined the association between viral load and CC severity, progression and development, whilst others have found that the amount of HPV DNA increases proportionally with lesion severity and can even be detected before cervical lesions develop [8-11]. However, other studies have found no such association [12-14].

As HPV-16 is the viral type most associated with cases of CC (50%–70%) [3,5], most knowledge concerning HPV viral load and CC has been based on HPV-16. Studies, which have included other HR-HPV types, have not led to comparable results regarding those obtained for HPV-16 [15,16].

The real-time polymerase chain reaction (RT-PCR) has been widely used and described in detecting and typing HPV, as well as quantifying a broad range of viral copies and normalising viral load according to the amount of human DNA, having high reproducibility, sensitivity, specificity and yield [13,17]. It was thus considered that it would provide a suitable approach for measuring HPV viral load, thereby facilitating investigating the role of HR-HPV viral load in developing CC [10,12,18].

The present study was thus aimed at using RT-PCR for determining the association between HPV viral load and the presence of CIN for six HR-HPV types, which have been previously reported as having the greatest prevalence in Colombia [19]. It was thus expected to contribute towards knowledge regarding the parameters leading to identifying HPV positive women having a higher risk of developing cervical lesions.

## Methods

### Study population and ethical considerations

Women eligible for the present study were voluntarily attending their cervical screening consultations between April 2007 and March 2010 in three Colombian regions (Girardot, Chaparral and Bogotá). Bogotá (the capital of Colombia) has the highest percentage of inhabitants, being mainly an urban population. Girardot is a city located in the Cundinamarca department which has focused its economy on the tourist sector due to its climate and infrastructure. The city of Chaparral (Tolima department) was included in the study as it is located in Colombia’s coffee-growing region and is also known for ecotourism. Girardot and Chaparral were grouped together in the “other city” category to improve the quality of the present study’s statistical analysis.

**Figure 1:**
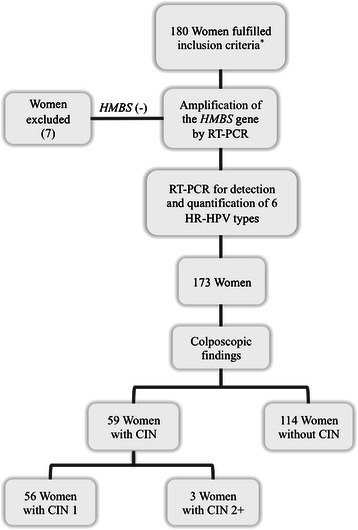
Flowchart of the studied population.^*^Inclusion criteria: women who had both a colposcopy result and HPV DNA detected by conventional PCR. RT-PCR: real-time polymerase chain reaction; HMBS: hydroxymethylbilane synthase gene; HR-HPV: high-risk human papillomavirus; CIN: cervical intraepithelial neoplasia; CIN 1: cervical intraepithelial neoplasia 1; CIN 2+: cervical intraepithelial neoplasia 2 or 3.

All the women signed a written informed consent form and completed a questionnaire regarding their sociodemographic characteristics, sexual behaviour and risk factor data before undergoing a gynaecological examination and providing a cervical smear. Samples were analysed using the Papanicolaou test and HPV DNA detection. Colposcopy and biopsy were performed in accordance with current Colombian screening programme guidelines, thereby establishing that women having normal, satisfactory cytology would continue following the 1-1-3 scheme, meaning that they should have a new control in a year’s time and, if this continued being normal, in three year’s time. However, colposcopy would be required when cytology was abnormal and, in case colposcopy was abnormal, samples would then be taken for pathology study, as in this study, for diagnosing CIN 1 and CIN 2+ [20]. Colposcopy and biopsy were also carried out for women having normal cytology but who were positive for HPV by conventional PCR, as previous studies have reported an increased risk of CIN 2+ development in women having normal cytology when they are HPV positive [21]. Due to biopsy not being taken from women having negative colposcopy, complete or satisfactory colposcopy (squamocolumnar junction completely visible), evaluation of the transformation area, having normal vascularisation and squamous, cylindrical epithelia without alterations were taken as criteria for guaranteeing the absence of lesions [22]. Colposcopy was chosen as the best method for defining the presence or absence of cervical lesions, as previous studies have found that colposcopy has a good correlation with histological results [23] and it remains the standard for detecting cervical lesions until new methods can be applied; in addition, cervical cytology has been reported worldwide as having variable sensitivity for detecting pre-neoplastic lesions and is considered a screening method which identifies women at risk of developing CC who must then be submitted to definitive diagnostic methods (colposcopy and biopsy) [20,24-26]. Women who had both a colposcopy result and HPV DNA detected by conventional PCR were thus included. Women were excluded in whom there was no amplification of the *Homo sapiens hydroxymethylbilane synthase* (HMBS) gene (Gene ID: 3145) by RT-PCR and those having an insufficient sample for analysis (Figure 1).

This study was supervised and approved by each institution’s Ethics Committee as follows: Fundación Instituto de Inmunología de Colombia’s Ethics Committee and the Ethics Committee of the Nuevo Hospital San Rafael E.S.E, Girardot, the Hospital San Juan Bautista de Chaparral E.S.E. Bioethics Committee and Hospital de Engativá (level II) Ethics Committee.

### HPV DNA collection, processing and detection by conventional PCR

Genomic DNA from cervical samples (stored at 4°C, in 95% ethanol) taken from HR-HPV 16, 18, 31, 33, 45 and 58 patients, which had been previously confirmed by conventional PCR (proving positive for at least one of the following previously described primers: GP5+/6+, MY09/11 or pU1M/2R) [27], was extracted using a Quick Extract DNA Extraction Solution kit (Epicentre, Madison, WI), according to the manufacturer’s recommendations. The samples were homogenised in 200 μL lysis buffer and incubated at 65°C for 6 minutes and then at 92°C for 2 minutes. The samples were then spun at 13,000 rpm for 10 minutes and the supernatant was stored at −20°C until use.

### Viral load quantification by RT-PCR

The methodology used in this study has already been described in detail in a previous article by our group [28]. Briefly, specific primers for each viral type and for *HMBS* were synthesised according to a study published by Moberg *et al.* [13]. The probes for each viral type and *HMBS* were designed, taking into account the types included in each reaction. Four parallel duplex real-time PCRs per patient were carried out (Table 1).

**Table 1:** **The probes and quenchers used for real-time polymerase chain reaction**

Test	Viral type	Size (bp)	Probe	Quencher
**Reaction 1**	HPV-16	78	FAM	ZEN/IBFQ
**Reaction 2**	HPV-18	80	Cy5	IBRQ
	HPV-31	78	HEX	ZEN/IBFQ
**Reaction 3**	HPV-33	78	FAM	ZEN/IBFQ
	HPV-45	76	Cy5	IBRQ
**Reaction 4**	HPV-58	109	HEX	ZEN/IBFQ
	*HMBS*	76	FAM	ZEN/IBFQ

Four parallel duplex real-time PCRs were performed per patient. Probe design for each viral type and *HMBS* was adjusted based on the types included in each reaction.

HPV: human papillomavirus; FAM: 6-carboxyfluorescein; Cy5: FluoroLink mono reactive dye Cy5; HEX: hexachlorofluoresceine; *HMBS*: hydroxymethylbilane synthase; ZEN/IBFQ: ZEN and Iowa Black FQ; IBRQ: Iowa Black RQ.

The cervical samples processed and identified as being HPV-positive by conventional PCR were used as template in PCR reactions for each fragment. The amplicons so obtained were purified with a Wizard PCR preps kit (Promega), once their quality has been evaluated on 3.25% agarose gel. A TOPO TA cloning kit was used for ligation, followed by transformation in TOP10 *E. coli* cells (Invitrogen). Several clones were incubated in LB broth and kept overnight (250 rpm at 37°C). Recombinant plasmids were purified using an UltraClean mini plasmid prep kit (MO BIO laboratories, California, USA) and sequenced using an automatic ABI PRISM 310 Genetic Analyser (PE Applied Biosystems, California, USA). Each insert’s integrity was checked by aligning the products with the respective theoretical sequenced fragments from each gene using Clustal W software [29].

#### Real-time PCR

Standardised RT-PCR assays with 10-fold serial plasmid dilutions (10^11^-10^6^copies) (using known DNA concentration and copy number) gave a standard curve for each viral type and the *HMBS* gene. CFX96 Touch RT-PCR detection system was used for analysis. Samples were tested for HPV-16, HPV-18, HPV-31, HPV-33, HPV-45 and HPV-58. The human *HMBS* gene was amplified in all samples to verify DNA integrity and determine viral copy number per cell. Four RT-PCR reactions were carried out per sample: HPV-16, HPV-18 and -31, HPV-33 and -45 and HPV-58 and *HMBS*. RT-PCR reaction conditions and protocols have been described previously [28].

Each run was performed in 96-well plates, including 6 standards for each viral type and *HMBS*, involving 10-fold plasmid dilutions (10^11^–10^6^ copy dynamic detection range) and a no template control to rule out DNA contamination.

The viral load was normalised to cellular DNA input using a previously described formula (Equation 1) [15]. Absolute and normalised viral loads were both log_10_ transformed.

Normalised viral load formula

1 $$ \mathrm{H}\mathrm{P}\mathrm{V}\kern0.5em \mathrm{D}\mathrm{N}\mathrm{A}\kern0.5em \mathrm{load}\left(\mathrm{H}\mathrm{P}\mathrm{V}\kern0.5em \mathrm{copies}/\mathrm{cell}\right)=\frac{\mathrm{Number}\kern0.5em \mathrm{of}\kern0.5em \mathrm{H}\mathrm{P}\mathrm{V}\kern0.5em \mathrm{copies}}{\left(\mathrm{Number}\kern0.5em \mathrm{of}\kern0.5em \mathrm{H}\mathrm{MBS}\kern0.5em \mathrm{copies}/2\right)} $$

### Statistical analysis

Sample size was calculated using the difference of proportions test for high viral load between women having and without cervical lesions (0.42 and 0.052 respectively) [8,30]; 0.05 significance, 90% statistical power and a 1:2 ratio between both groups were established. This meant that at least 23 women with lesions and 46 women without them were required for the study. Based on the availability of women without CIN, two women without cervical lesions reported by colposcopy were matched to each woman with CIN by age (within 5 years) and date of enrolment. As only a limited amount of women had CIN 2+ or high-grade squamous intraepithelial lesions (CIN 2+, according to The Bethesda System (TBS)), CIN category was established which included women having CIN 2+ and women with CIN 1 or low-grade squamous intraepithelial lesions (CIN 1, according to TBS) [31,32] to improve the quality of the present study’s statistical analysis.

Analysis was based on type-specific HPV infection rather than on individual women, taking into account that multiple infection is common in the Colombian population [19].

Categorical variable differences between groups were assessed by Chi-squared test or Fisher’s test, as appropriate, using a 0.05 significance level. Median and interquartile ranges (IQR) were used for quantitative variables, according to the data distribution.

HPV DNA load distribution between women according to colposcopy and biopsy results was analysed by the Mann–Whitney *U* test or Kruskal Wallis test, depending on the number of groups to be compared. Both absolute HPV DNA load and normalised HPV DNA load were analysed. Absolute viral load was categorised according to percentile distribution in both groups of patients as follows: negative ≤ 0, low 0 < VL ≤10^5^ HPV copies and high >10^5^ HPV copies (to ensure better quality analysis).

Considering that women with CIN were paired with women without CIN by age and date of entering the study, conditional logistic regression was used for assessing the association between the HPV DNA load for each viral type and cervical lesion frequency according to colposcopy results. This analysis was not done taking the presence of biopsy-defined cervical lesions as outcome, as histology results were not available for all patients included in the study. Crude odds ratio (OR) and adjusted OR with their 95% confidence intervals (CI) were estimated, taking control variables into account, such as origin, ethnicity, age on starting to have sexual relations and the number of infecting HPV types. Hypothesis testing involved a two-tailed test (0.05 significance); STATA 10 was used for all statistical analysis.

## Results

180 patients fulfilled the inclusion criteria; 7 of them were excluded from statistical analysis, as their *HMBS* gene could not be amplified. This meant that 114 women were classified as negative for intraepithelial lesions (92.98% having normal cytology) and 59 women having CIN identified by colposcopy (56 women having CIN 1 and 3 having CIN 2+) were included in the analysis (Figure 1).

According to the diagnostic algorithm, a biopsy was taken from 59 women having colposcopy-defined cervical lesions; however, results were only obtained for 45 women as the samples taken for pathology regarding the remaining 14 women were unsatisfactory or had been lost. 23.73% (n = 14) of the women had confirmation of CIN 1 by biopsy (only one woman with CIN 2+ was found). Two of the CIN 2+ women detected by colposcopy had CIN 1 by biopsy.

Regarding women with CIN, median age was 40 years old (14 years IQR) and 41.5 years old (13 years IQR) in women without CIN. Most women participating in the study came from the city of Girardot (60.69%; n = 105); 76.19% (n = 80) of these women were negative for lesions. 95.95% of the women in the study were mestizos (n = 166) and the remaining percentage (4.05%) was made up of indigenous, white and black women. The distribution of socio-demographic characteristics and risk factors associated with CC and the detection of HPV infection was compared between both groups (those with CIN and those without it), significant differences being found regarding origin (p < 0.05) (Table 2).

**Table 2:** **The distribution of socio-demographic characteristics and risk factors**

Characteristic	Categories	n	%	With CIN (n = 59)		Without CIN (n =114)		p
				n	%	n	%	
**Age, years**	**<30**	29	16.76	11	18.64	18	15.79	0.493
	**30–40**	54	31.21	21	35.59	33	28.95	
	**>40**	90	52.02	27	45.76	63	55.26	
**Origin**	**Bogotá**	65	37.57	32	54.24	33	28.95	**0.001**
	**Other city**	108	62.43	27	45.76	81	71.05	
**Ethnicity**	**Other**	7	4.05	3	5.08	4	3.51	0.691
	**Mestizo**	166	95.95	56	94.92	110	96.49	
**Average monthly income** ^*^	**≤ minimum**	155	89.06	53	89.83	102	89.47	0.942
	**>minimum**	18	10.40	6	10.17	12	10.53	
**Educational level**	**No schooling**	1	0.58	1	1.69	0	0.00	0.094
	**Primary**	82	47.40	22	37.29	60	52.63	
	**Secondary**	74	42.77	28	47.46	46	40.35	
	**Technical**	10	5.78	6	10.17	4	3.51	
	**Graduate**	6	3.47	2	3.39	4	3.51	
**Marital status**	**Single**	17	9.83	4	6.78	13	11.40	0.673
	**Married**	20	11.56	7	11.86	13	11.40	
	**Divorced**	8	4.62	4	6.78	4	3.51	
	**Living with partner**	126	72.83	43	72.88	83	72.81	
	**Widow**	2	1.16	1	1.69	1	0.88	
**Healthcare scheme affiliation**	**Subsidised- linked**	159	91.91	52	88.14	107	93.86	0.191
	**Contributory-private**	14	8.09	7	11.86	7	6.14	
**Smoker**	**No**	146	84.39	49	83.05	97	85.09	0.726
	**Yes**	27	15.61	10	16.95	17	14.91	
**Age at first intercourse, years**	**<16**	41	23.70	10	16.95	31	27.19	0.133
	≥**16**	132	76.30	49	83.05	83	72.81	
**Lifetime number of sexual partners**	**1**	72	41.62	26	44.07	46	40.35	0.868
	**2–3**	84	48.55	27	45.76	57	50.00	
	**>3**	17	9.83	6	10.17	11	9.65	
**Contraceptive method**	**None**	65	37.57	19	32.20	46	40.35	0.697
	**Surgery**	52	30.06	15	25.42	22	19.30	
	**Hormonal**	19	10.98	18	30.51	34	29.82	
	**Barrier**	37	21.39	7	11.86	12	10.53	
**Pregnancies**	**None**	4	2.31	1	1.69	3	2.63	0.326
	**1–2**	76	43.93	28	47.46	48	42.11	
	**3–4**	74	42.77	27	45.76	47	41.23	
	**>4**	19	10.98	3	5.08	16	14.04	
**Abortions**	**None**	82	47.40	27	45.76	55	48.25	0.818
	**1**	68	39.31	25	42.37	43	37.72	
	**≥2**	23	13.29	7	11.86	16	14.04	
**STD**	**No**	137	79.19	47	79.66	90	78.95	0.913
	**Yes**	36	20.81	12	20.34	24	21.05	

Values in bold = p < 0.05.

^*^The minimum average monthly income (2014 rate) would be roughly US $300.

p = p value; CIN: cervical intraepithelial neoplasia; STD: sexually transmitted disease.

Overall, 91.91% (n = 159) of the sample proved positive for the detection of HPV by RT-PCR, i.e. 93.22% (n = 55) of women with CIN (92.86% positive from the group having CIN 1 and 100% positive from the group having CIN 2+) and 91.23% (n = 104) of women without lesions. 79.24% (n = 126) of all infected women were infected by more than one viral type; this was observed in 81.82% (n = 45) of women with CIN and 77.88% (n = 81) of women negative for lesions. Simultaneous infection was more frequent concerning 2 high-risk viral types in women without lesions (n = 29; 27.88%) and 3 types in women with cervical lesions (n = 19; 34.54%). The most frequently encountered viral types were HPV-18 and HPV-16 in multiple infections, in both groups.

The type-specific distribution revealed HPV-18 as being most frequent in both groups (69.49% in women having CIN and 66.66% in women without CIN), followed by HPV-16 (57.63%) and HPV-45 (38.98%) in women having lesions and HPV-16 (45.61%), HPV-31 (45.61%) and HPV-45 (38.60%) in women proving negative for lesions. HPV-33 had the lowest infection frequency in both groups.

Higher high viral load was recorded concerning HPV-18, HPV-16 and HPV-33 infection in women with CIN, whilst high viral load was most frequent in HPV-31, HPV-45 and HPV-58 infection in women without lesions (Table 3).

**Table 3:** **Type-specific HR-HPV viral load distribution by category**

HPV type	n	%	With CIN (n = 59)						Without CIN(n = 114)						p
			Negative		Low viral load		High viral load		Negative		Low viral load		High viral load		
			n	%	n	%	n	%	n	%	n	%	n	%	
**HPV-16**	86	49.71	25	42.37	12	20.34	22	37.29	62	54.39	13	11.40	39	34.21	0.186
**HPV-18**	117	67.63	18	30.51	10	16.95	31	52.54	38	33.33	18	15.79	58	50.88	0.928
**HPV-31**	71	41.04	40	67.80	1	1.69	18	30.51	62	54.39	3	2.63	49	42.98	0.257
**HPV-33**	14	8.09	54	91.53	0	0.00	5	8.47	105	92.11	1	0.88	8	7.02	0.846
**HPV-45**	67	38.73	36	61.02	9	15.25	14	23.73	70	61.40	10	8.77	34	29.82	0.366
**HPV-58**	56	32.37	42	71.19	7	11.86	10	16.95	75	65.79	16	14.04	23	20.18	0.772
**HR-HPV** ^*^	159	91.91	4	6.78	8	13.56	47	79.66	10	8.77	12	10.53	92	80.70	0.777

HPV DNA load: categorised as ≤ 0 = negative. 0 < VL ≤ 10^5^ HPV copies = low viral load. >10^5^ HPV copies = high viral load.

^*^HR-HPV: high risk-human papillomavirus, infection by at least one high-risk viral type from the 6 analysed here.

HPV: human papillomavirus; CIN: cervical intraepithelial neoplasia; p = p value.

**Figure 2:**
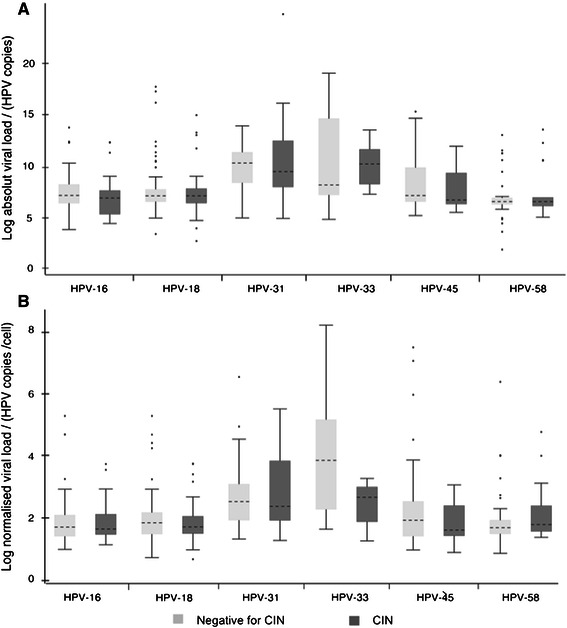
**Distribution of viral load for 6 HR-HPV types in both groups of patients. A.** Absolute viral load. **B** Normalised viral load. The dotted line indicates the median; the box represents the interquartile range (IQR). The whiskers extending from the boxes are the upper and lower limits. Diamond markers represent extreme values. No statistically significant differences were observed regarding DNA load distribution of each HPV type between both groups of patients (Mann–Whitney *U* test). CIN: cervical intraepithelial neoplasia.

Figures 2 shows absolute (A) and normalised (B) viral load distribution for each HR-HPV type, comparing both groups of women. It is worth stating that HPV-31 (in women without CIN) and HPV-33 (in women having CIN) were the HR-HPV viral types having the highest absolute viral load (median = 9.41 (2.58 IQR) HPV copies for HPV-31 and median = 9.33 (2.94 IQR) HPV copies for HPV-33) whilst HPV-58 infection had the lowest absolute viral load in both groups of women. The range of values for normalised viral load was lower than for absolute (up to 10^8^ HPV copies). The highest absolute viral load was detected for HPV-31 in women with CIN (10^22^ HPV copies) and highest normalised viral load for HPV-33 in women without CIN. No statistically significant differences were observed regarding viral load distribution (absolute and normalised) for each HR-HPV type in either group of patients.

The three patients having CIN 2+ were positive for HR-HPV; HPV-18 and HPV-31 were detected in two of them, whilst the other one was positive for HPV-18, HPV-16 and HPV-45. Even though women having CIN 2+ had a higher viral load (normalised for HPV-18 and absolute for HPV-16) than women having CIN 1, the differences in viral load distribution were not statistically significant. However, normalised viral load for HPV-31 was greater in women negative for cervical lesion and having CIN 1 compared to women having CIN 2+ (marginal significance, i.e. p = 0.052).

The distribution of viral load was also analysed for each HR-HPV type, according to biopsy result. Similar results were found to those with colposcopy (i.e. higher absolute viral loads in women having a severer degree of lesion); and for some types (HPV-31, HPV-33 and HPV-58) higher normalised viral loads; however, the differences were not statistically significant due to the amount of women analysed (Table 4).

**Table 4:** **Distribution of 6 HR-HPV types’ viral load regarding biopsy results**

Viral type	Negative (n = 28)			CIN 1 (n = 16)			CIN 2+ (n = 1)		
	% (n)	Viral load, median (IQR)		% (n)	Viral load, median (IQR)		% (n)	Viral load, median (IQR)	
		Absolute	Normalised^*^		Absolute	Normalised*		Absolute	Normalised^*^
**HPV-16**	66.67 (22)	6.42 (1.69)	1.79 (0.54)	57.89 (11)	6.77 (3.04)	1.69 (0.64)	0	n/a	n/a
**HPV-18**	66.67 (22)	6.29 (1.34)	1.84 (0.51)	68.42 (13)	6.61 (2.28)	1.67 (1.79)	100 (1)	7.02 (n/a)	2.07 (n/a)
**HPV-31**	30.30 (10)	8.51 (1.90)	2.39 (0.38)	31.58 (6)	9.69 (6.00)	3.50 (2.17)	0	n/a	n/a
**HPV-33**	3.03 (1)	6.75 (n/a)	1.98 (n/a)	10.53 (2)	8.48 (1.70)	2.37 (2.06)	100 (1)	10.57 (n/a)	3.13 (n/a)
**HPV-45**	51.52 (17)	6.13 (2.95)	1.79 (1.00)	42.11 (8)	6.24 (1.17)	1.61 (0.80)	0	n/a	n/a
**HPV-58**	21.21 (7)	5.93 (3.89)	2.14 (2.35)	36.84 (7)	6.12 (0.34)	1.75 (0.28)	0	n/a	n/a
HR-HPV**	94.34 (31)	6.37 (1.20)	2.06 (0.63)	94.74 (18)	6.77 (2.97)	2.12 (1.37)	100 (1)	8.80 (n/a)	2.60 (n/a)

Absolute and normalised viral loads were both log_10_ transformed.

^*^HPV copies/cell = number of HPV copies/(number of *HMBS* copies/2).

^**^HR-HPV: high risk-human papillomavirus, infection by at least one high-risk viral type from the 6 analysed here.

HPV: human papillomavirus; CIN: cervical intraepithelial neoplasia; CIN 1: cervical intraepithelial neoplasia 1; CIN 2+: cervical intraepithelial neoplasia 2 or 3; n/a: not applicable.

Crude and adjusted odds ratios (OR) were calculated for estimating the magnitude of absolute viral load association with CIN for each viral type. The conditional logistic regression model revealed that HPV-16 infection was significantly associated with greater frequency regarding cervical lesions. However, lesions occurred more frequently in the group of women having low viral load for HPV-16 (0 < VL ≤ 5.86 HPV copies) than in women having a high load (>5.86 HPV copies), (3.53 OR_a_, 1.16–10.74 95%CI; 2.63 OR_a_, 1.09–6.36 95%CI, respectively). It was also found that CIN frequency was lower in women having HPV-31 and high viral load (>5.14 HPV copies; 0.34 OR_a_, 0.15–0.78 95%CI). No significant associations were obtained for the other viral types with the presence of CIN (Table 5).

**Table 5:** **Conditional logistic regression model**

HPV type	Viral load	With CIN / without CIN	Crude OR (95%CI)	Adjusted OR^*^	95%CI
**HPV-16**	**Negative**	25/62	*Reference*		
	**0 < VL ≤ 5.86**	12/13	2.19 (0.88–5.43)	**3.53**	**1.16**–**10.74**
	**5.86 < VL**	22/39	1.27 (0.64–2.50)	**2.63**	**1.09**–**6.36**
**HPV-18**	**Negative**	18/38	*Reference*		
	**0 < VL ≤ 5.95**	10/18	1.14 (0.45–2.89)	1.72	0.52–5.69
	**5.95 < VL**	31/58	1.06 (0.52–2.17)	1.77	0.68–4.63
**HPV-31**	**Negative**	40/62	*Reference*		
	**0 < VL ≤ 5.14**	1/3	0.52 (0.04–6.29)	0.15	0.01–2.26
	**5.14 < VL**	18/49	0.60 (0.32–1.14)	**0.34**	**0.15**–**0.78**
**HPV-33**	**Negative**	54/105	*Reference*		
	**0 < VL ≤ 4.60**	0/1	0.00 (0 - .)	0	0 - .
	**4.60 < VL**	5/8	1.43 (0.45–4.50)	1.67	0.44–6.28
**HPV-45**	**Negative**	36/70	*Reference*		
	**0 < VL ≤ 5.98**	9/10	1.53 (0.60–3.92)	2.94	0.92–9.44
	**5.98 < VL**	14/34	0.79 (0.38–1.67)	1.13	0.43–2.96
**HPV-58**	**Negative**	42/75	*Reference*		
	**0 < VL ≤ 5.97**	7/16	0.83 (0.32–2.11)	0.73	0.23–2.31
	**5.97 < VL**	10/23	0.83 (0.37–1.83)	0.86	0.35–2.12
**HR-HPV** ^**^	**Negative**	4/10	*Reference*		
	**0 < VL ≤ 5.94**	8/12	1.73 (0.40–7.47)	1.01	0.23–4.50
	**5.94 < VL**	47/92	1.18 (0.35–4.00)	1.39	0.25–7.81

Values in bold = p < 0.05.

^*^Adjusted for origin, ethnicity, age at first intercourse and number of viral types.

^**^HR-HPV: high-risk-human papillomavirus, infection by at least one high-risk viral type from the 6 analysed here (viral load = sum of viral loads of HPV types detected/ number of HPV types detected.

HPV: human papillomavirus; CIN: cervical intraepithelial neoplasia; VL: viral load; OR: odds ratio.

## Discussion

This study involved using RT-PCR; this enabled type-specific evaluation of the viral load of the most frequently occurring oncogenic types in Colombia (HPV-16, -18, -31, -33, -45 and -58) [19] for determining each type’s association with precursor lesions of CC. As the method has high sensitivity, specificity and has a broad dynamic range of viral detection (up to 10^22^ HPV copies) this provided the best approach for this study [12,13,16,18,33].

More HPV infections were found in women having CIN in our sample, amongst whom all women having CIN 2+ were HPV positive. The foregoing was consistent with the fact that almost 99.7% of CC cases are associated with HPV [1]. Previous studies have demonstrated that HPV prevalence in women having CIN is high, proportionally increasing as lesion severity increases [30,34,35]. The prevalence found here was greater than that reported in the literature (100% in CIN 2+, 92.86% in CIN 1 and 91.23% in women without CIN). Women were included in this study who had been previously identified as HPV positive using conventional PCR; this explained the high prevalence of HPV when using RT-PCR in women without lesions. However, variable infection prevalence in women without CIN has been found worldwide (mean = 12.6%) [35,36].

Multiple infection frequency has been variable (16.3%–55%) in previous reports concerning women having lesions [35]; up to 3.4% infection by multiple types of HR-HPV has been described in women without lesions [37]. The present study revealed more multiple infections (in both the general population and women having CIN and those without them) regarding previous reports worldwide, but similar to that previously reported in Colombia [27,38]. However, RT-PCR was used which has high sensitivity and allows small amounts of viral DNA to be detected, compared to other methods [13,18]. This has been previously demonstrated by studies carried out involving RT-PCR which have reported high multiple infection frequency [39,40]. Such differences regarding co-infection prevalence reported in various studies might have been due to their design, sample size, the HPV detection methods used and the population being studied (geographic, demographic and clinical factors) [37].

HPV-18 and HPV-16 occurred most frequently in the present study, followed by HPV-45 and HPV-58. Differences concerning type-specific prevalence have been reported according to geographic and demographic factors [3,35]. It is worth noting that the two most common types found here are responsible for the 70% of cases of CC [41] and that the HPV genotypes evaluated in this study have been reported amongst the 8 HR-HPV types most frequently occurring around the world, in both women without lesions and women with CC [2,3,35].

Absolute viral load was highest in women having CIN compared to women without lesions determined by both colposcopy and biopsy; an increase in the viral load was observed for HPV-18 and HPV-33 proportional to the degree of injury. The foregoing was consistent with previous studies which have revealed the effect of viral load on developing CC. Most HPV-16 studies have found that viral load has increased in relation to the degree of cervical lesion severity [8-11,15,16,42].

An association between viral load and cervical lesion frequency (as assessed by colposcopy) was observed in this study just for HPV-16 and HPV-31. The present study’s results highlighted the fact that women having low HPV-16 load (<5.86 HPV copies) had higher cervical lesion frequency. Such results agreed with those from a study by Manawapat, Stubenrauch *et al.*, [43] which showed that women having persistent HPV-16 infection had lower viral load than those who had a transient infection (4.72 copies/cell *cf* 20 copies/cell; p = 0.0003). It has been found recently that low viral load was characteristic of intermittently detected persistent infection [44]. Reduced viral load has been described in women having CIN; this has been explained by HPV genome integration associated with down-regulation of viral DNA synthesis, thereby affecting immune system activation and thus reducing the probability of infection being eliminated [43,45-47]. Accordingly, a long period of latency accompanied by low viral load would probably be observed, representing a greater risk for infection persistence and lesion progression [48].

Contrary to our findings regarding HPV-16 viral load, the present study found that a high HPV-31 load (>5.14 HPV copies) was associated with lower cervical lesion frequency. As mentioned previously regarding HPV-16 results, it has been shown that viral load has been greater in transitory infections regarding patients having persistent infection [43]. This agreed with the finding that clearance of HPV-16 infection has been preceded by a transient viral load peak or a plateau phase [33]; such high load was probably necessary for the immunological system to become induced, thereby favouring HPV elimination. According to the above, HPV-31 infections are probably transitory and such association is mediated by an immune system response to high viral load which can eliminate the infection and thus CC precursor lesions do not progress or such lesions regress spontaneously [47].

Regarding the other viral types (HPV-18, -33, -45 and -58), no association was found between viral load and cervical lesion frequency; such result was supported by data from other authors [14-16,42,49,50]. However, a study by Moberg, Gustavsson *et al.*, found that high HPV-16, HPV-31 and HPV-18/45 viral load increased the risk of developing carcinoma *in situ* (CIS) [51].

The pertinent literature gives different cut-off points when categorising viral load, depending on the quantification technique used (RT-PCR, Hybrid Capture II (HCII)) [8] and distribution in a particular population being evaluated [9,51]. A study which evaluated the clinical significance of HPV-16 and -18 viral loads determined that HPV-16 viral load was related to cervical lesion severity, having a 3.0×10^6^ copies/million cells threshold, this being highly specific for grade 2 diagnosis [15]. Taking the foregoing into account, viral load was categorised in the present study according to percentile distribution, leaving 10^6^ copies as cut-off point for ensuring analysis quality.

It is worth stressing that this technique managed to detect a broad range of viral load, even after stratifying by colposcopy result and viral type. However, this hampered establishing viral load cut-off points to enable identifying women at greater risk of developing cervical lesions; previous studies have also experienced such difficulty [12,16,33].

This work’s value lies in it being a study where a reproducible, sensitive and specific technique (i.e. RT- PCR) was used for detecting and quantifying viral load (absolute and normalised) not just for one viral type but for the 6 most frequently occurring high-risk HPV types described to date in Colombia. Besides, this is the first study carried out in Colombia which has included women from regions having high HPV infection prevalence and which was aimed at evaluating the association between HPV viral load and cervical lesion frequency.

This study’s results were obtained from a single evaluation of HPV viral load; this means that predicting the risk of lesion progression and developing CC later on cannot be ascertained from this. However, it can be stated that our results were consistent with some findings reported in longitudinal studies [33,43,44,48]. The infection duration time of the women included in this study was also unknown; HPV-16 might thus have been greater in women having CIN and lower in HPV-31 women. Another limitation of this study was the low number of women having CIN 2+ which hindered generalising the results to all CC precursor lesions. An analysis of HPV viral load dynamics could thus be more reliable and provide more information for estimating whether HPV infection will worsen or clear and predicting the development of CC or cervical lesions. Prospective studies on women having HPV infection which would include type-specific determination (according to local prevalence) of viral load and women having cervical lesions with different degrees of severity are thus needed for confirming our results.

## Conclusions

A significant association was found in this study, low HPV-16 and high HPV-31 viral loads were associated with higher CIN frequency; this might have been related to infection duration and immune system response. HPV infection’s effect on developing CC is influenced by viral load, meaning that measuring load could improve the predictive value of HPV detection; however, the scope of quantification depends on the viral type being detected. These findings support the idea of quantifying viral load (as a type-specific marker of CC), coupled to cytology, for improving and strengthening CC screening programmes. This would lead to identifying HPV positive women at greater risk of developing cervical lesions, as well as identifying women as yet lacking cervical anomalies for predicting the beginnings of neoplasia.

## Abbreviations

HPV: Human papillomavirus
HR-HPV: High-risk human papillomavirus
CC: Cervical cancer
CIN: Cervical intraepithelial neoplasia
CIN 1: Cervical intraepithelial neoplasia 1
CIN 2+: Cervical intraepithelial neoplasia 2 or 3
HMBS: Hydroxymethylbilane synthase
PCR: Polymerase chain reaction
RT-PCR: Real-time polymerase chain reaction
DNA: Deoxyribonucleic acid
VL: Viral load
STD: Sexually-transmitted diseases
HC II: Hybrid capture II
FAM: 6-carboxyfluorescein
Cy5: FluoroLink mono reactive dye Cy5
HEX: hexachlorofluoresceine
ZEN/IBFQ: ZEN and Iowa Black FQ
IBRQ: Iowa Black RQ
SD: Standard deviation
CI: Confidence interval
IQR: Interquartile range
n/a: Not applicable
OR: Odds ratio
